# Improvement of Dynamic On-Resistance in GaN-Based Devices with a High-Quality In Situ SiN Passivation Layer

**DOI:** 10.3390/mi14061227

**Published:** 2023-06-10

**Authors:** Jeong-Gil Kim, Jun-Hyeok Lee, Dong-Min Kang, Jung-Hee Lee

**Affiliations:** 1 Terrestrial & Non-Terrestrial Integrated Telecommunications Research Laboratory, Electronics and Telecommunications Research Institute, Daejeon 34129, Republic of Korea; jeonggil.kim@etri.re.kr (J.-G.K.); kdm1597@etri.re.kr (D.-M.K.); 2 GaN Part, DB-Hitek, Eumseong 27605, Republic of Korea; junhyeok.lee@dbhitek.com; 3 L&D Inc., Daegu 34138, Republic of Korea

**Keywords:** AlGaN/GaN, MISHEMT, in situ SiN, dynamic on-resistance, breakdown voltage

## Abstract

In this paper, we compared the characteristics of normally-on/off AlGaN/GaN MISHEMTs passivated by an in situ/ex situ SiN layer. The devices passivated by the in situ SiN layer revealed enhanced DC characteristics, such as the drain current of 595 mA/mm (normally-on) and 175 mA/mm (normally-off) with the high on/off current ratio of ~10^7^, respectively, compared with those of the devices passivated by the ex situ SiN layer. The MISHEMTs passivated by the in situ SiN layer also exhibited a much lower increase of dynamic on-resistance (R_ON_) of 4.1% for the normally-on device and 12.8% for the normally-off device, respectively. Furthermore, the breakdown characteristics are greatly improved by employing the in situ SiN passivation layer, suggesting that the in situ SiN passivation layer can remarkably not only suppress the surface-trapping effects, but also decrease the off-state leakage current in the GaN-based power devices.

## 1. Introduction

Recently, there has been enormous progress in the field-effect transistors (FET) with the wide band-gap semiconductors. GaN-based devices have demonstrated excellent performances for high-power/frequency applications due to their high breakdown electric field, high saturation velocity, and high thermal stability [1,2]. Especially, GaN-based metal-insulator-semiconductor high-electron-mobility transistors (MISHEMTs) have demonstrated a high performance of the devices in the power-switching applications [3,4,5]. MISHEMTs can reduce the gate leakage dramatically and enlarge gate voltage swing. In addition, the GaN-based normally-off devices are also attractive candidates for the power-switching applications [6,7,8]. However, GaN-based devices still have the demand of reducing the trapping effects from the surface and buffer layers, which results in the current dispersion and higher dynamic on-resistance. In particular, the traps on the surface can capture the electrons and then act as a virtual gate, which depletes 2-DEG (2-dimensional electron gas) channel electrons and increase on-resistance simultaneously [9]. There have been many efforts to reduce the surface-trapping effect and passivate the surface with SiN, SiO_2_, and Al_2_O_3_ deposited by low-pressure chemical vapor deposition (LPCVD), plasma-enhanced chemical vapor deposition (PECVD), and atomic layer deposition (ALD), respectively [10,11,12,13,14]. Moreover, new studies of improving the interface have been introduced such as the insertion of graphene, an AlNO layer deposited by plasma-enhanced atomic layer deposition (PEALD), and a SiON layer deposited by the PECVD, respectively [15,16,17]. However, a high density of interface trap states (D_it_) in the order of ~10^13^–10^14^ cm^−2^ eV^−1^ are still usually present at the dielectric/AlGaN barrier interface with these ex situ passivation layer [18,19,20,21]. 

Compared with these ex situ deposited passivation layers, a high-quality in situ SiN passivation layer can be grown at a much higher temperature in a metal-organic chemical vapor deposition (MOCVD) chamber immediately after the growth of the AlGaN barrier, without exposing the barrier to the ambient air to eliminate the process-induced surface contamination and/or damage which results in less formation of the interface trap states [22,23,24]. In addition, the in situ SiN layer is grown at the relatively high growth temperature and low growth rate, resulting in a much better dielectric quality [25,26,27].

In this work, we present the effects of an in situ SiN passivation layer on the device performance, including DC characteristics, pulsed I-V characteristics, and breakdown characteristics. We fabricated two different AlGaN/GaN-MISHEMTs with the in situ SiN passivation layers; one for the normally-on HEMT utilizing the in situ SiN layer as the gate insulator, and the other for the gate recessed normally-off HEMT with removing the in situ SiN layer in the recessed gate region. The dynamic on-resistances (R_ON_) of the fabricated devices were remarkably improved due to the effective suppression of the trapping effects.

## 2. Materials and Methods

### 2.1. Epitaxial Structure

**Figure 1:**
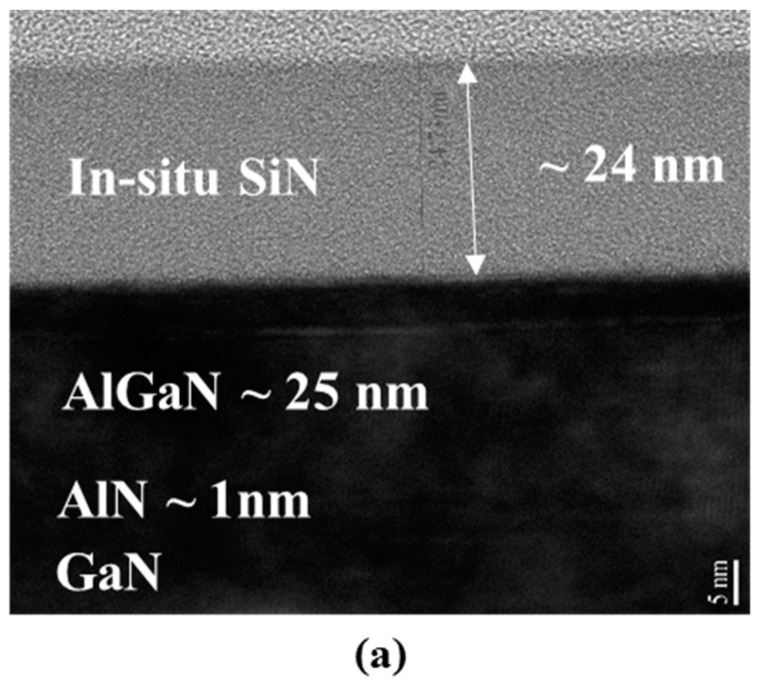

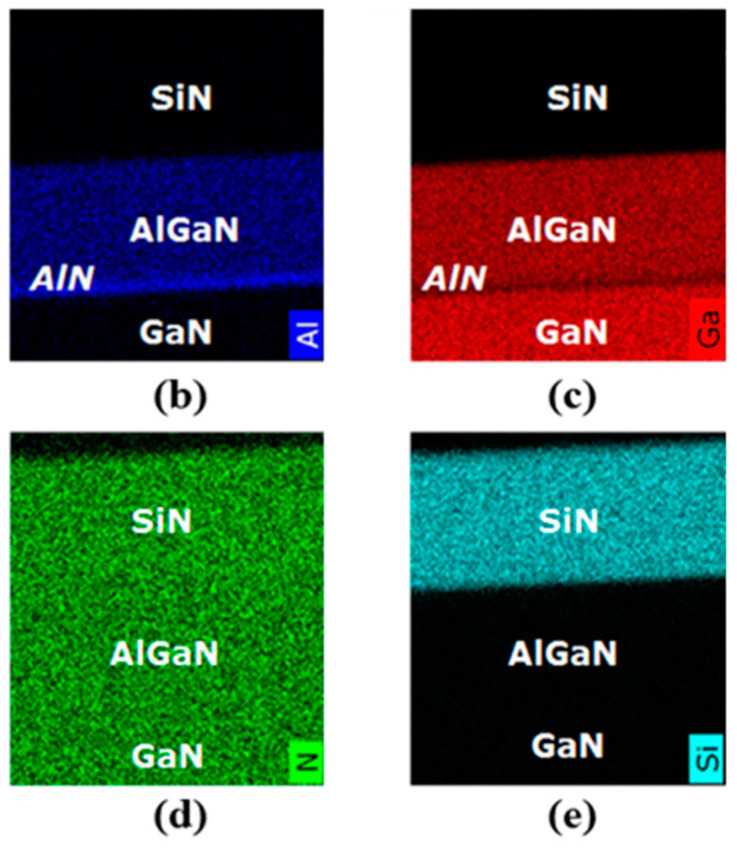
(**a**) Cross-sectional TEM image of the AlGaN/GaN heterostructure with the in situ SiN and EDX analysis of (**b**) Al, (**c**) Ga, (**d**) N, and (**e**) Si, respectively.

In situ SiN/AlGaN/GaN heterostructures were grown on 2 inch SiC substrates. Trimethylgallium (TMGa), trimethylaluminum (TMAl), ammonia (NH3), and silane (SiH_4_) were used as the sources of Ga, Al, N, and Si, respectively. Firstly, a 50 nm-thick initial GaN nucleation layer was grown at a low temperature of 525 °C, and then a 2 µm-thick C-doped semi-insulating GaN buffer layer was grown at 1060 °C, which consists of the 1st GaN layer grown with a pressure of 50 Torr and the 2nd GaN layer with 300 Torr. Sequentially, an AlGaN/GaN heterostructure consisting of a 50 nm-thick unintentionally doped GaN channel layer, and a 25 nm-thick Al_0.25_Ga_0.75_N barrier were grown in the same growth temperature of 1060 °C. To improve the 2-DEG property, a 1 nm-thick AlN layer was inserted between the barrier and the GaN channel layer. Finally, an in situ SiN layer was grown at 1000 °C. The cross-sectional TEM image and the energy-dispersive X-ray spectroscopy (EDX) analysis shown in Figure 1, indicate that the thickness of the in situ SiN layer being 24 nm, and the interface between the in situ SiN layer and AlGaN barrier being abrupt in nature.

Prior to device fabrication, the two-dimensional electron gas (2-DEG) property was evaluated by Hall measurements using the Van der Pauw method, such as the sheet concentration of 8.9 × 10^12^ cm^−2^, electron mobility of 1950 cm^2^/V·s, and sheet resistance of 357 ohm/sq, respectively.

### 2.2. Device Fabrication

**Figure 2:**
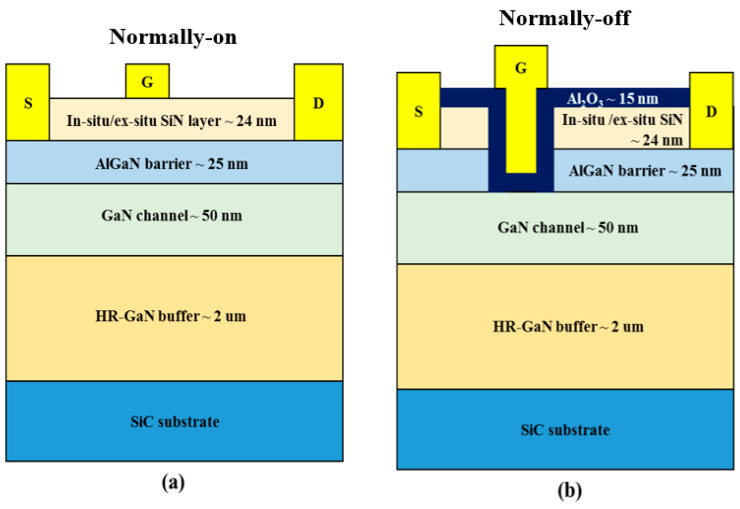
Schematic representation of (**a**) normally-on and (**b**) normally-off AlGaN/GaN MISHEMTs passivated with in situ/ex situ SiN layer.

As shown in Figure 2, normally-on and normally-off devices were fabricated to assess the effect of the in situ SiN passivation layer. For the fabrication of normally-on devices of MISHEMTs shown in Figure 2a, the mesa isolation, which is one of the most widely used techniques for device isolation was first performed using Cl_2_ gas in an inductively coupled-plasma reactive ion etcher (ICP-RIE). For the protection of the epitaxial layer and preventing the increase of sheet resistance during the rapid thermal annealing (RTA) process, a 30 nm-thick SiN protection layer was deposited by PECVD. An ohmic metal stack consisting of Si/Ti/Al/Ni/Au (1/25/160/40/100 nm) was then deposited by an e-beam evaporator, followed by 2-step rapid thermal annealing at 500 °C for 20 s and 850 °C for 30 s in N_2_ ambient to form the source/drain ohmic contact. The contact resistance was evaluated as 0.6 ohm-mm measured by the transfer length method (TLM). Finally, Ni/Au (50/150 nm) was deposited for the gate electrode.

For the fabrication of normally-off MISHEMTs shown in Figure 2b, the in situ SiN layer and the AlGaN barrier was sequentially recess-etched in the gate region using dry etching in CF_4_ and Cl_2_ gas by ICP-RIE, respectively. Then, a 15 nm-thick ALD Al_2_O_3_ layer was deposited as a gate insulator. The gate length (L_G_), the gate width (W_G_), and the gate–drain distance (L_GD_) of the fabricated devices were 3, 100, and 5 u **µ** m, respectively. For comparison of the device characteristics, ex situ SiN/AlGaN/GaN normally-on/off MISHEMTs were also fabricated. The thickness of the ex situ SiN layer was approximately 25 nm.

## 3. Results and Discussion

**Figure 3:**
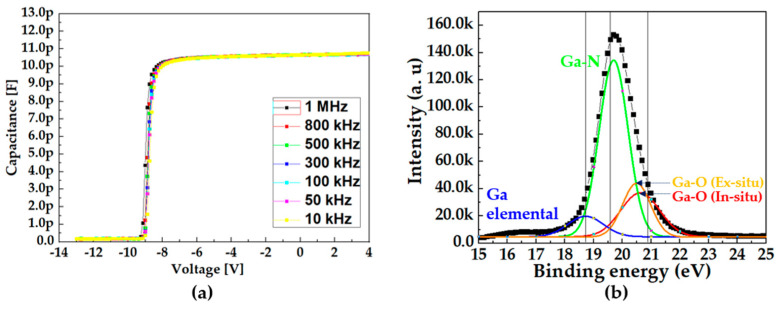
(**a**) Multi-frequency C–V characteristics. (**b**) XPS analysis of the in situ SiN passivation layer.

Figure 3a shows the C–V characteristics measured from the metal/in situ SiN/AlGaN/GaN stack capacitor with varying frequencies from 10 kHz to 1 MHz at room temperature. The C–V curves revealed a sharp transition and negligible frequency dispersion, indicating that the in situ SiN layer deposited on the AlGaN barrier forms a very high-quality interface. 

The extracted *D_it_* at E_c_ − E_T_ = 0.3 eV was calculated as 3.66 × 10^11^ eV^−1^ cm^−2^ Equation (1) below, indicating that the In situ SiN layer effectively passivates the AlGaN surface, and reduces the interface trap density at the dielectric/barrier compared to that with the ex situ SiN layer [28,29].

(1) $$D_{it}=\frac{C_{ox}}{q}\left(\frac{dV_{G}}{d\psi_{s}}-1\right)-\frac{C_{s}}{q}=\frac{C_{ox}}{q}\frac{dV_{G}}{d\psi_{s}}$$

X-ray photoelectron spectroscopy (XPS) was performed to compare the density of the Ga-O bonds, which are believed to be main origin of the surface trap. In Figure 3b, black line shows the XPS analysis of the in situ SiN passivation layer and it consists of three components with binding energies of 20.8, 19.6, and 18.7 eV were extracted, which corresponded to Ga-O, Ga-N, and the Ga element, respectively. The intensity of the Ga-O bond decreased when the AlGaN surface was covered with the in situ SiN passivation layer, as shown in Figure 3b. Consequently, it was expected that the in situ SiN passivation layer effectively suppressed the oxygen incorporation at the AlGaN surface to reduce the trapping effects at the device surface as the in situ SiN layer grew immediately after the AlGaN barrier was grown in the MOCVD chamber.

**Figure 4:**
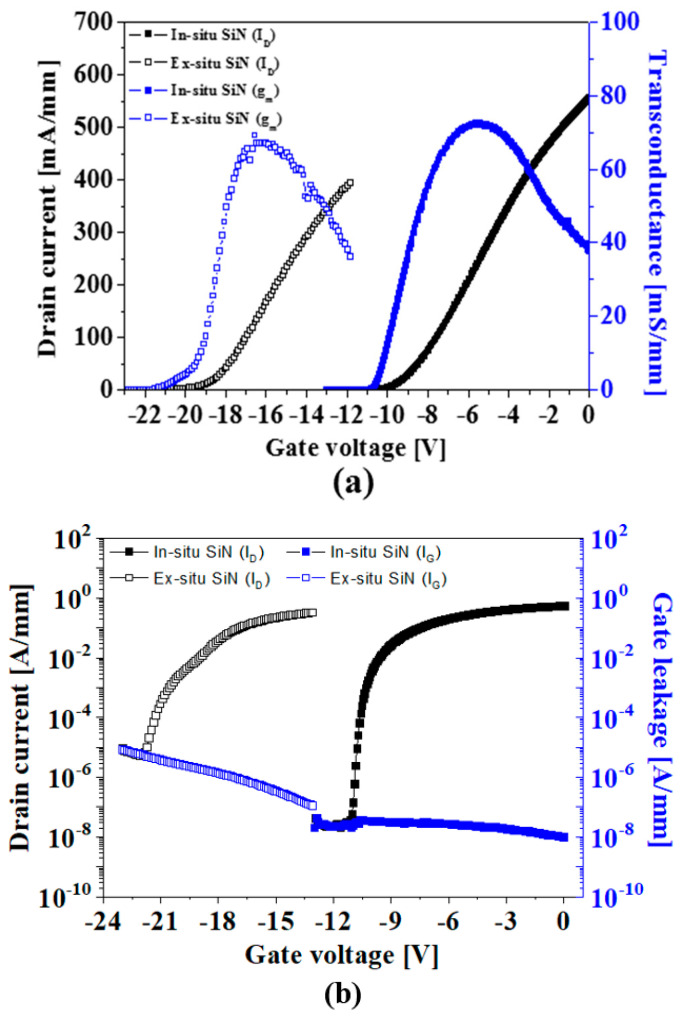

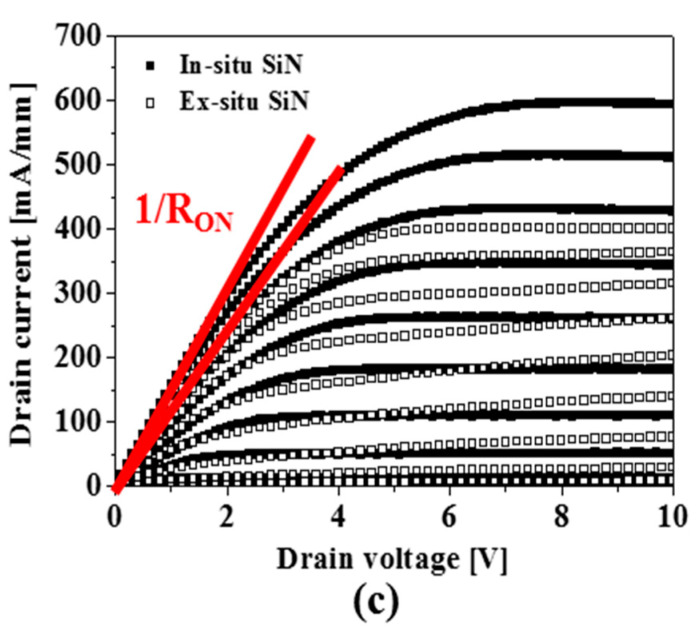
Comparison of the normally-on AlGaN/GaN MISHEMTs in transfer characteristics, including (**a**) linear scale, (**b**) log scale, and (**c**) output characteristics.

The transfer I–V and DC output characteristics of both AlGaN/GaN MISHEMTs with an in situ/ex situ SiN layer were evaluated. Figure 4a shows the transfer characteristics of the normally-on AlGaN/GaN MISHEMTs with an in situ/ex situ SiN layer at the saturation region (V_DS_ = 10 V). The MISHEMTs passivated with an ex situ SiN layer showed the threshold voltage of −18.5 V, the drain current of 401 mA/mm at VG = −12 V, and the peak transconductance of 67 mS/mm, respectively. The MISHEMTs passivated with the in situ SiN layer showed improved device characteristics, such as the drain current of 595 mA/mm at V_G_ = 0 V, the threshold voltage of −9.2 V, and the peak transconductance of 76 mS/mm, respectively. The large negative threshold voltage for the device with the ex situ SiN passivation layer was believed to be due to the high density of the positive fixed charges in the ex situ SiN layer, which also increased the 2DEG density to satisfy the charge neutrality.

Moreover, the device passivated by the in situ SiN showed a very low off-state leakage current of ~2 × 10^−8^ A/mm and a high on/off current ratio of ~10^7^ compared to those of the device passivated with the ex situ SiN layer as shown in Figure 4b. It is believed that in situ SiN effectively prevents the generation of the Ga-O bond at the AlGaN surface, and hence suppresses the surface-trapping effects and the surface leakage current, resulting in the low off-state leakage current.

Figure 4c compares the output characteristics of the AlGaN/GaN MISHEMTs passivated by the in situ/ex situ SiN layer. The device passivated with the in situ SiN layer showed a relatively low R_ON_ of 5.6 ohm-mm extracted in the linear region and at V_G_ where the maximum drain current flows. Meanwhile, the device passivated with the ex situ SiN layer showed a higher R_on_ of 8.4 ohm-mm. This was believed to be due to the gate lag caused by the surface trapping [30].

**Figure 5:**
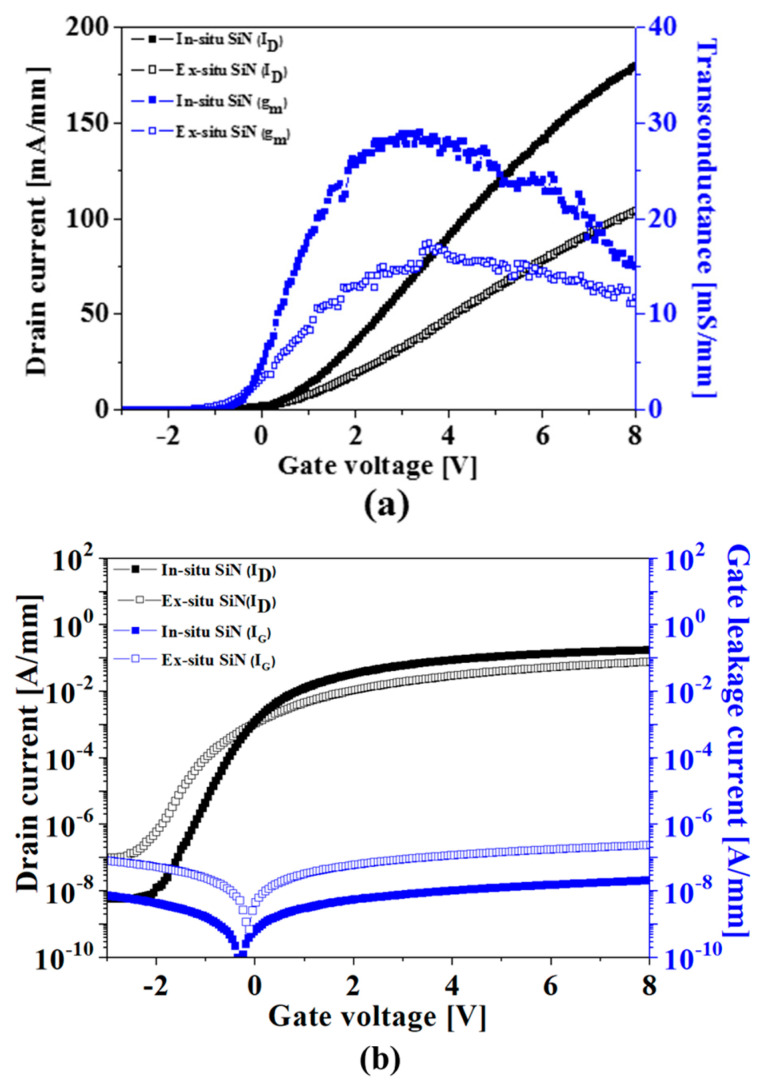

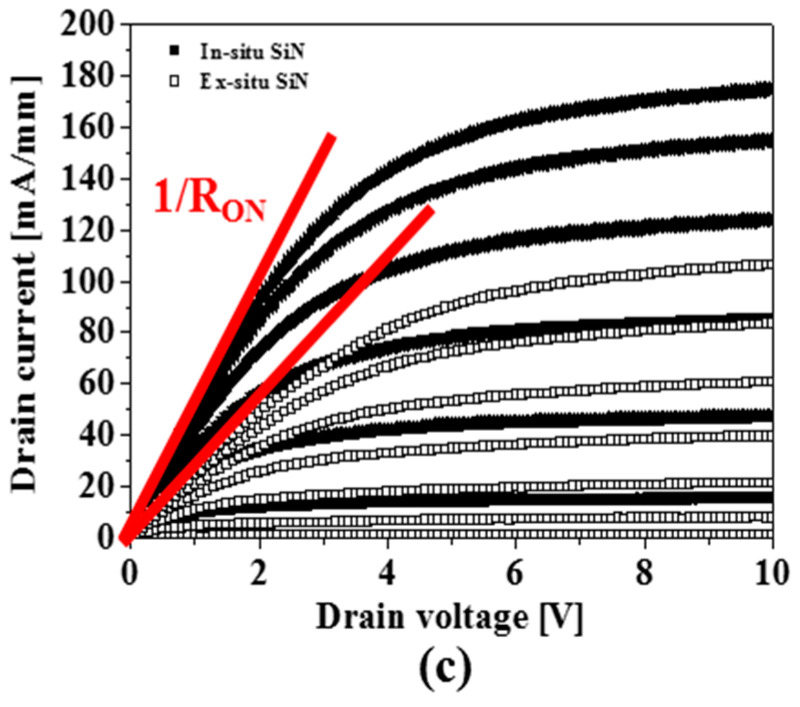
Comparison of the normally-off AlGaN/GaN MISHEMTs in transfer characteristics, including the (**a**) linear scale, (**b**) log scale, and (**c**) output characteristics.

The merit of the in situ SiN layer was also proven in the normally-off MISHEMTs as shown in Figure 5. The device passivated with the in situ SiN layer showed a higher drain current of 175 mA/mm at V_G_ = 8 V and a peak transconductance of 29 mS/mm, compared to that of the drain current of 104 mA/mm and the peak transconductance of 17 mS/mm for the device containing the ex situ SiN layer, respectively. Furthermore, the device with the in situ SiN layer exhibited a much improved off-state leakage current of 6 × 10^−9^ A/mm with a high on/off current ratio of ~10^7^ and R_ON_ of 25 ohm-mm compared to the values of the off-state leakage current of 1 × 10^−7^ A/mm and R_ON_ of 41 ohm-mm for the devices passivated with the ex situ SiN layer, respectively.

**Figure 6:**
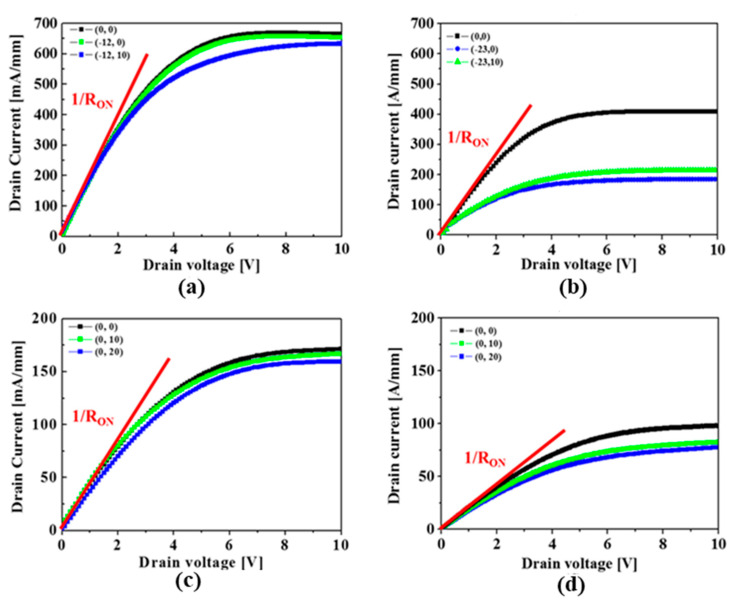
Pulsed I-V characteristics of the normally-on MISHEMTs passivated by the (**a**) in situ and (**b**) ex situ SiN layers, and of the normally-off MISHEMTs passivated by the (**c**) in situ and (**d**) ex situ SiN layers.

The pulsed I–V characteristics were measured under the condition of a pulse width of 50 μs and period of 1 mS, respectively, as shown in Figure 6. The devices passivated with the in situ SiN layer showed a much lower percentage increase of dynamic R_on_ of 4.1% for the normally-on device, and 12.8% for the normally-off device, respectively, while the devices with passivated with the ex situ SiN layer revealed a corresponding percentage increase of dynamic R_on_ of 50 and 16.9%, respectively. Therefore, it was concluded that the in situ SiN passivation layer effectively suppressed the surface-trapping effects.

**Figure 7:**
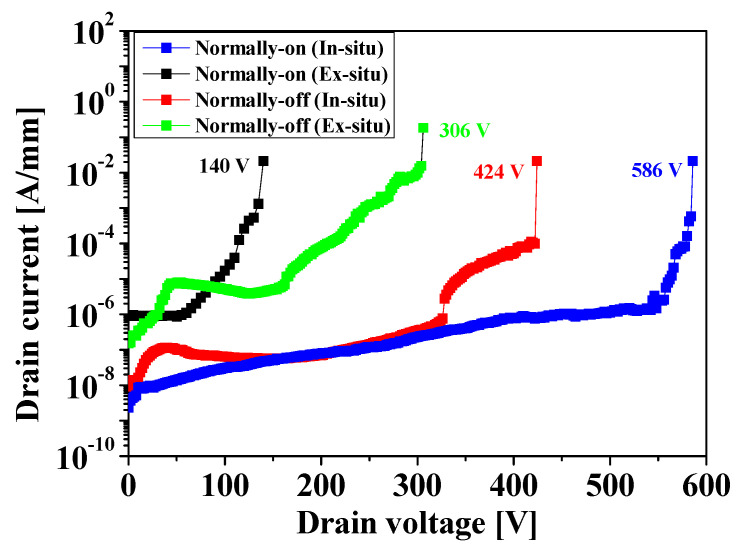
Comparison of the off-state breakdown characteristics in the normally-on/off MISHEMTs passivated by the in situ/ex situ SiN layer.

The breakdown characteristics of the devices with a L_GD_ of 5 um were measured using the Keithley model 248 high voltage supply and a Keithly 6485 picoammeter, as shown in Figure 7. The normally-on/off MISHEMTs passivated with the in situ SiN layer exhibited a much higher breakdown voltage of 586 and 424 V, respectively, while the devices passivated with ex situ SiN layer exhibited a relatively poor breakdown voltage of 140 and 306 V, respectively, indicating that the in situ SiN passivation layer can remarkably suppress the surface-trapping effects in the GaN-based devices.

## 4. Conclusions

The characteristics of the normally-on/off AlGaN/GaN MISHEMTs passivated with an in situ/ex situ SiN layer were investigated. The devices passivated with the in situ SiN layer showed much improved DC characteristics, such as the drain current of 595 mA/mm (normally-on) and 175 mA/mm (normally-off) with the high on/off current ratio of ~10^9^, respectively, compared to those of the devices passivated with the ex situ SiN layer. The devices passivated with the in situ SiN layer showed a much lower percentage increase of dynamic R_on_ of 4.1% for the normally-on device and 12.8% for the normally-off device, respectively. 

It was also found that the in situ SiN passivation layer greatly improved the breakdown characteristics for the device of 586 and 424 V for normally-on and normally-off, respectively, indicating that the passivation with the high-quality in situ SiN layer devices can withstand much higher voltages, thereby improving its robustness.

In conclusion, employing an in situ SiN passivation layer in AlGaN/GaN MISHEMTs leads to improved DC characteristics, including higher drain currents, increased on/off current ratios, lower dynamic R_on_, and enhanced breakdown characteristics. These findings have implications for the design and optimization of high-performance GaN-based transistors.

## Data Availability

Data available in a publicly accessible repository.
